# Supplementary material for: Splice-Junction-Based Mapping of Alternative Isoforms in the Human Proteome
Source: Cell Rep. Author manuscript; Available in PMC 2020 Jan 15. (PMC6961840; doi:10.1016/j.celrep.2019.11.026)
Supplement: 2 [file NIHMS1546469-supplement-2.zip › DF1/PXD006675/CardiacFibroblast/CardiacFibroblast_19_FXYD5_AGFHEDDPFFYDEH.pdf]

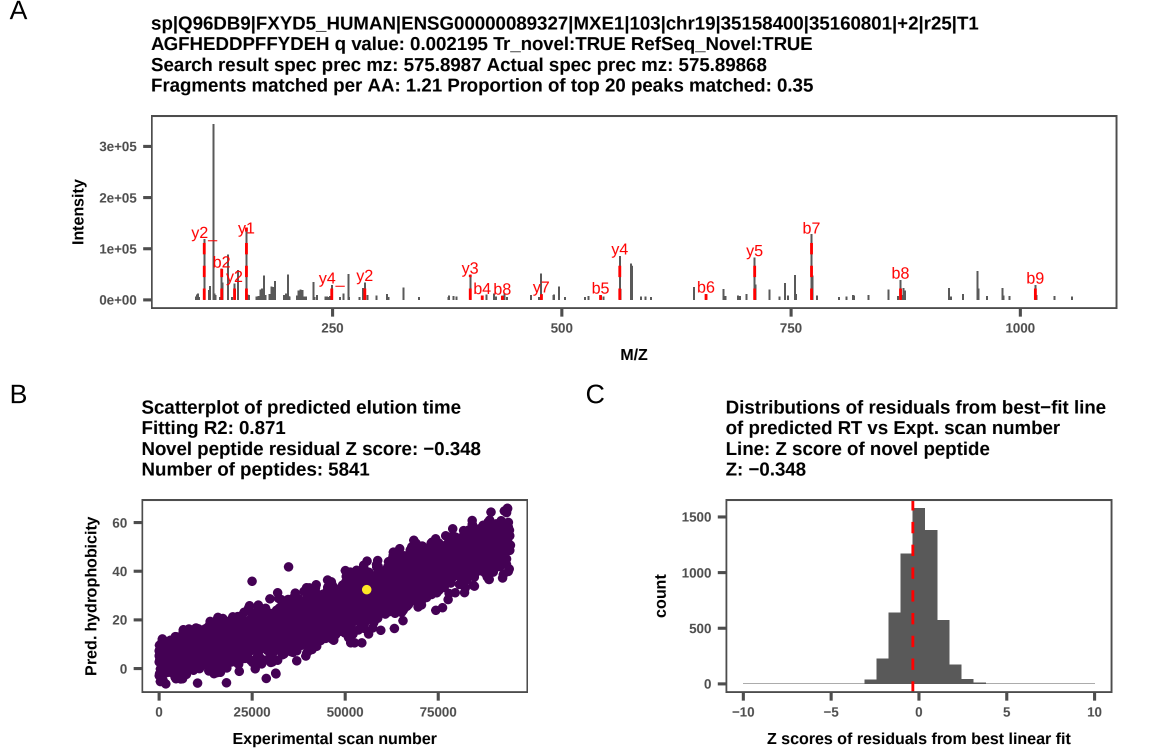

A
sp|Q96DB9|FXYD5_HUMAN|ENSG00000089327|MXE1|103|chr19|35158400|35160801|+2|r25|T1
AGFHEDDPFFYDEH q value: 0.002195 Tr_novel:TRUE RefSeq_Novel:TRUE
Search result spec prec mz: 575.8987 Actual spec prec mz: 575.89868
Fragments matched per AA: 1.21 Proportion of top 20 peaks matched: 0.35
Intensity
3e+05
2e+05
1e+05
0e+00
y2_
y1
b2
y2
y4_
y2
y3
b4
b8
y7
b5
y4
b6
y5
b7
b8
b9
250
500
750
1000
M/Z
B
Scatterplot of predicted elution time
Fitting R2: 0.871
Novel peptide residual Z score: −0.348
Number of peptides: 5841
Pred. hydrophobicity
60
40
20
0
0
25000
50000
75000
Experimental scan number
C
Distributions of residuals from best−fit line
of predicted RT vs Expt. scan number
Line: Z score of novel peptide
Z: −0.348
count
1500
1000
500
0
−10
−5
0
5
10
Z scores of residuals from best linear fit
